# Postoperative pain of single-visit endodontic treatment with gutta-percha versus MTA filling: a randomized superiority trial

**DOI:** 10.1186/s12903-023-03372-6

**Published:** 2023-12-19

**Authors:** Masoud Khabiri, Sahel Kamgar, Pedram Iranmanesh, Abbasali Khademi, Mahmoud Torabinejad

**Affiliations:** 1 Department of Endodontics, Faculty of Dentistry, Isfahan (Khorasgan) Branch, Islamic Azad University, Isfahan, Iran; 2 Department of Endodontics, Isfahan (Khorasgan) Branch, Islamic Azad University, Isfahan, Iran; 3 Department of Endodontics, Dental Research Center, Dental Research Institute, School of Dentistry, Isfahan University of Medical Sciences, Isfahan, Iran; 4 School of Dentistry, Loma Linda University, Loma Linda, CA USA; 5 School of Dentistry, Isfahan University of Medical Sciences, Hezar-Jerib Ave, Isfahan, 81746-73461 Iran

**Keywords:** Asymptomatic apical periodontitis, Gutta-percha, Mineral trioxide aggregate, Pain

## Abstract

**Background:**

Postoperative pain has remained a challenge for clinicians. This randomized superiority trial compared the levels of postoperative pain following the use of gutta-percha (GP) and sealer or mineral trioxide aggregate (MTA) as root canal filling materials in teeth with asymptomatic apical periodontitis.

**Methods:**

A total of 119 patients were initially evaluated in this two-arm, parallel-group, single-blind, superiority randomized trial. The inclusion criteria were participants aged 18–65 years with single-canal premolars diagnosed with asymptomatic apical periodontitis. The participants were finally divided into two groups using the permuted block randomization method. In the GP group (N = 46), the cleaned and shaped root canals were filled with gutta-percha and AH Plus sealer, while in the MTA group (N = 48), the cleaned and shaped root canals were filled with an MTA apical filling and a coronal gutta-percha and sealer. Patient pain level was measured 6, 12, 24, 48, and 72 h postoperatively using a 10-point visual analog scale (VAS). The data were analyzed by the chi-square, independent t, Friedman, and Mann-Whitney U tests.

**Results:**

The mean of VAS scores decreased significantly over time in both groups (P < 0.001). The mean VAS scores were significantly lower in the MTA filling group than in the other group (P < 0.05). Female patients reported higher VAS scores at 6- and 12-hour periods in both groups (P < 0.05).

**Conclusion:**

MTA as a root canal filling material might be a valuable option for clinicians due to its low postoperative pain.

**Trial registration:**

The trial protocol was registered at the Registry of Clinical Trials (IRCT20191104045331N1).

**Supplementary Information:**

The online version contains supplementary material available at 10.1186/s12903-023-03372-6.

## Background

Postoperative pain following root canal treatment has remained a challenge for dental clinicians [1]. The frequency of patients suffering postoperative pain following root canal treatment ranges from 3 to 58% [2]. Postoperative pain is caused by inflammation of periapical tissues in response to debris, bacteria [3], and dental materials [4]. During the inflammation, several immune mediators such as prostaglandins, histamine, Hageman factor, clotting cascade, fibrinolytic system, and complement system are involved and cause swelling and pain [5].

After the chemo-mechanical debridement of the root canal, the root canal filling materials are used to seal the disinfected canal. Ideally, root canal filling materials should be easy to handle, nonirritating for periapical tissues, radiopaque, bacteriostatic, and should not shrink after application [6]. Gutta-percha (GP) and sealer, which have been extensively used for years and demonstrated most characteristics of an ideal material for root canal filling material [7]. However, their disadvantages include the inability to bond to dentinal walls [7], the inability to reinforce the tooth structure [8], and not being entirely removed in retreatment cases [9]. Furthermore, all commonly used sealers show some degree of toxicity, which decreases over time [10]. To address these drawbacks, novel materials such as calcium silicate-based cement have been introduced.

Bioceramics are biocompatible and bioactive materials that possess optimal dimensional stability, antibacterial activity [11], and slight expansion and provide proper seal [12]. These materials release calcium hydroxide, induce hard tissue formation [13], are currently commercially available, and may enhance endodontic treatments [14]. Mineral Trioxide Aggregate (MTA) is the most well-known bioceramic material that has most of the aforementioned essential properties [13] and has been used in endodontic procedures such as root canal filling material [15–18] and sealer [19] with desirable clinical outcomes [20].

To the best of our knowledge, there is no research evaluating the role of root canal filling materials on endodontic postoperative pain. Although some animal models indicated that the MTA induces some analgesic effects and reduces nerve activity [4, 21, 22], there is no clinical study in this regard. The purpose of this randomized superiority trial was to compare the levels of postoperative pain following the use of GP and sealer, or MTA as root canal filling materials in teeth with asymptomatic apical periodontitis. The alternative hypothesis was that postoperative pain would be lower in the single-visit endodontic treatment with GP than with MTA plug in participants with asymptomatic apical periodontitis.

## Methods

The present randomized superiority trial was registered in the Registry of Clinical Trials (IRCT20191104045331N1) on 27/10/2020, available at https://www.irct.ir/trial/46195. The ethics committee of The Azad University - Isfahan (Khorasgan) Branch, approved the study (IR.IAU.KHUISF.REC.1398.075), and all of the participants in this trial signed informed consent forms. All methods were performed in accordance with the declaration of Helsinki. The present trial was reported using the *CONSORT 2010 checklist*.

The trial patients were selected from those referred to the Endodontics Department of the School of Dentistry. The inclusion criteria consisted of participants aged 18–65 years that had restorable single-canal maxillary or mandibular premolar teeth with asymptomatic apical periodontitis (no sensitivity to percussion or palpation) and necrotic pulp confirmed using ENDO-FROST spray cold (Coltène-Whaledent, Langenau, Germany) and periapical radiography (periapical index of 3, 4, or 5) without any preoperative pain. The exclusion criteria included retreatment cases, teeth with a periapical index of 1 or 2, and patients taking an analgesic during the past 12 h.

Initially, a total of 119 participants that required endodontic treatment were included in this randomized, single-blind, two-arm parallel, superiority trial. With a 2-sided alpha risk of 0.05, a sample size of 44 subjects in each group is a prerequisite to distinguish a significant difference of 0.5 (20%) in VAS score with a power of 80 [23]. To compensate for any causes of attrition such as loss during follow-up, the sample size was increased by 15%. Consequently, a sample size of 50 was scheduled for each group. At the beginning of the study, based on our inclusion and exclusion criteria, 100 eligible patients were selected and randomly allocated to either the GP group (N = 50) or the MTA group (N = 50) using the permuted block randomization method (block size of 4). To conceal the allocations, the sequentially numbered cards were given to participants via opaque, sealed envelopes by a person not involved in the randomization process (AK). A senior investigator was responsible for recruitment and randomization (MK).

In each group, after the administration of an inferior alveolar nerve block using Lidocaine HCl 2% with Epinephrine 1:100000 (DarouPakhsh, Tehran, Iran), each tooth was isolated using a rubber dam (Sanctuary, Perak, Malaysia), and the access cavity was prepared. If the pulp was diagnosed with partial necrosis, the participant was excluded and replaced. Following coronal flaring by a #SX ProTaper Universal (Dentsply Sirona, Ballaigues, Switzerland), a Root ZX Apex Locator (Morita, Tokyo, Japan) was used to determine the working length (indicator reach 0.5 according to manual), which was confirmed by digital radiography (0.5 mm short of the radiographic apex). After the negotiation of canals with K-file #15 and 20 (Mani, Tochigi, Japan), canals were prepared by #S1, S2, F1, F2, and F3 ProTaper Universal rotary files using the crown-down technique. So, the apical preparation size for each group was 0.30 mm. Apical patency was maintained by using a K-file #10. Sodium hypochlorite 2.5% (Cerkamed, Stalowa Wola, Poland) was used for irrigation, and sterile saline (DarouPakhsh, Tehran, Iran) was used as the final irrigating solution using a 27-gauge side-vented needle (Ultradent, South Jordan, UT) which was placed in the apical third of the canal during irrigation (total quantity of 6 mL of sodium hypochlorite and 30 mL of sterile saline was used) [24]. The canals were then dried with paper points (Diadent, Chungcheongbuk-do, Korea). Following root canal preparation, the root of each group was filled with either GP or MTA.

In the GP group, after checking the master apical cone with a digital periapical radiograph (Vatech, Gyeonggi-do, Korea), the root canals were filled with GP (Meta Biomed, Chungcheongbuk-do, Korea) and AH-26 sealer (Dentsply, Tulsa Dental, Tulsa, OK) using the traditional lateral compaction technique. Sealer was prepared with AH Plus Jet mixing syringe (Dentsply DeTrey, Konstanz, German) and placed on canal orifices with intra-oral tips (Dentsply DeTrey, Konstanz, German). Applying the Master-Point Technique, the wall of the canal was wet gently by counterclockwise rotation of the point.

In the MTA group, the root canals were filled with an MTA apical filling followed by a coronal GP and sealer filling. ProRoot MTA powder (Dentsply Tulsa Dental, Tulsa, OK) was mixed with the liquid at a 3:1 ratio according to the manufacturer’s instructions. An MTA filling with 3–5 mm thickness was placed using a Map system (Produits Dentaires SA, Vevey, Switzerland) and packed using a plugger (Dentsply, Maillefer, Switzerland). After confirming the quality and length of MTA filling by radiographic, the remaining root canal was filled with GP and AH-26 sealer using the lateral compaction technique.

At the end of the session, temporary restoration (Coltosol F, Altstatten, Switzerland) was applied and the final radiograph was obtained for each group; if the final root canal filling lengths exceeded the working length, the participant was excluded and replaced. Finally, the patients were referred to the Restorative Department for placement of the final restoration. The entire procedure was performed in a single-visit by a 5-year experienced practitioner (S.G) using 3.5x magnification. During each procedure, the participants were blinded to the obturation method. The flowchart of the experimental procedure is presented in Additional file 1: Figure S1.

To assess the severity of postoperative pain, the participants were instructed to rate their pain level 6, 12, 24, 48, and 72 h after treatment using a questionnaire containing a 10-point Visual Analog Scale (VAS). The questionnaires were then collected after three days by an investigator (S.G). The VAS was classified using the following scale [25]: no pain (0); mild pain (1–3); moderate pain (4–6), and severe pain (7–10). Patients also received a rescue bag containing 10 tablets of Ibuprofen 400 mg (DarouPakhsh, Tehran, Iran) with instructions in case of severe pain. Any adverse effect was also evaluated after the follow-up period.

The data were fed into IBM SPSS Statistics, Version 20.0 (Armonk, NY: IBM Corp.). The independent t-test and chi-square test were used to compare the difference between the two groups in terms of age, gender, and tooth location. The Friedman test was used to compare pain intensity over time. The Mann-Whitney U test was run to compare pain levels between the groups, genders, and tooth locations. The significance level was set at 0.05.

## Results

**Fig. 1:**
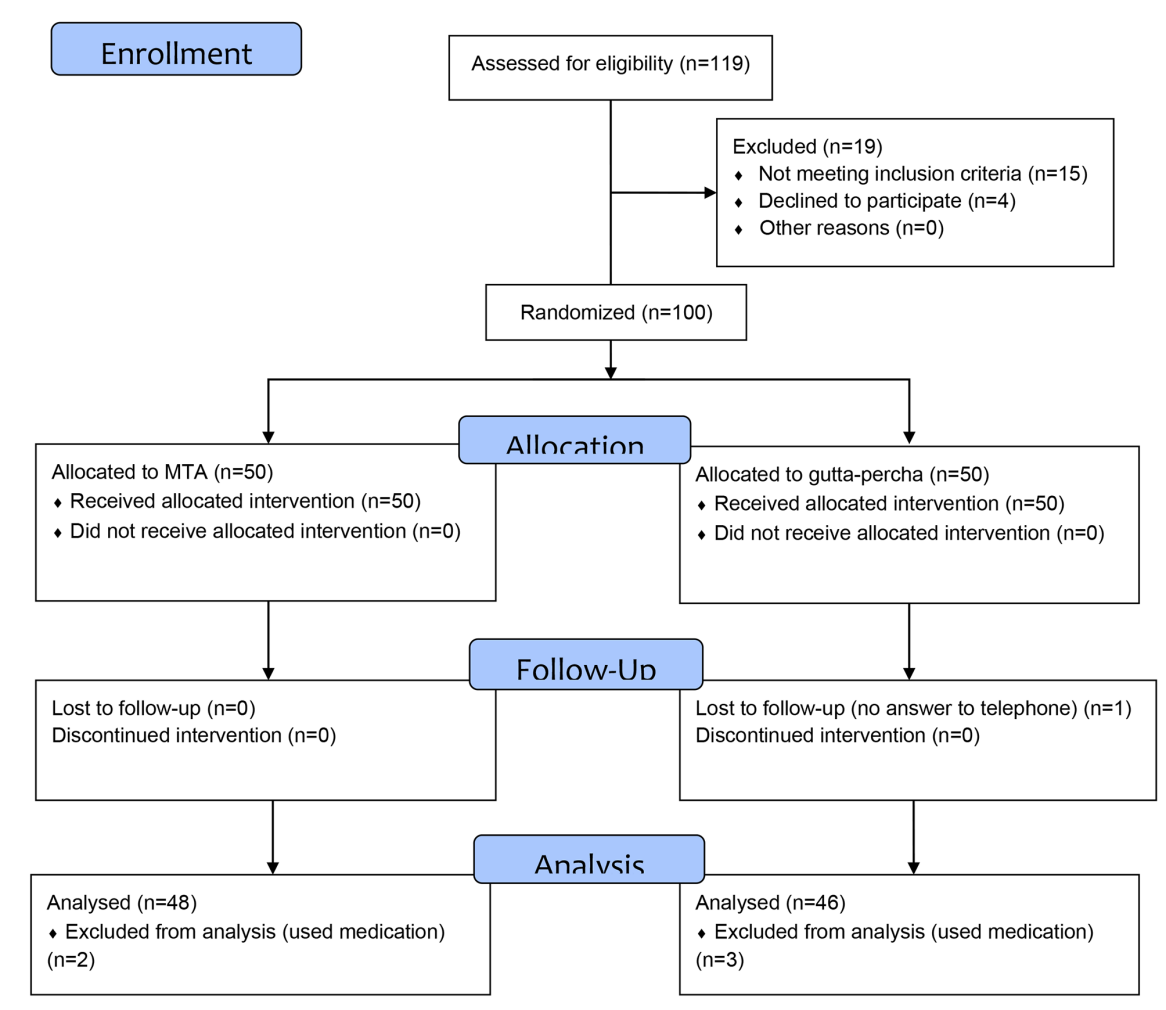
CONSORT 2010 Flow Diagram

One participant was excluded from the GP group for not returning the required datasheet within the 3-day follow-up period. Two participants in the MTA group and three participants in the GP group were also excluded because they did not follow the study instructions. Thus, the data were collected from a total of 94 patients, 46 in the GP group and 48 in the MTA group (Fig. 1).

The demographic and clinical characteristics of the two groups were not significantly different in the initial stage of the trial (Table 1). The pain level distribution based on the groups is shown in Additional file 1: Table S1. Maximum pain intensity was noted after 6 h, which decreased significantly over time in both groups (P < 0.001, Friedman test). The mean VSA scores were significantly lower in the MTA filling group than in the GP group samples filled with GP and sealer at all-time points (P < 0.05, Mann-Whitney U test) (Table 2).

**Table 1:** Baseline demographic and clinical features of both groups

	Gutta-percha n = 50	MTA n = 50	P-value
**Age** **(years)**			P > 0.05^1^
Mean (SD)	37.30 (12.28)	37.42 (11.12)	
Range	18–58	18–58	
**Gender [n (%)]**			P > 0.05^2^
Female	37 (74)	33 (66)	
Male	13 (26)	17 (34)	
**Jaw of premolar [n (%)]**			P > 0.05^2^
Maxilla	33 (66)	32 (64)	
Mandible	17 (34)	18 (36)	

SD: Standard deviation; ^1^t-test, ^2^Chi-square

**Table 2:** Mean (SD) of pain intensity at different time points for both groups

Time point	Gutta-percha	MTA	P-value^1^
	Mean (SD)	Mean (SD)	
**6 h**	2.74 (2.93)	1.04 (2.43)	< 0.001
**12 h**	2.30 (2.89)	0.84 (2.42)	< 0.001
**24 h**	1.86 (2.76)	0.58 (2.04)	< 0.001
**48 h**	1.68 (2.74)	0.40 (1.65)	0.002
**72 h**	1.44 (2.60)	0.38 (1.65)	0.005
**P-value** ^**2**^	< 0.001	< 0.001	

SD: Standard deviation; ^1^Mann–Whitney U test; ^2^Friedman test

In terms of gender, the female patients reported higher VAS scores in both groups after 6 and 12 h (P < 0.05, Mann-Whitney U test), but not at other time intervals. Splitting the groups by tooth location resulted in no significant difference between the maxillary and mandibular teeth in both groups (P > 0.05, Mann-Whitney U test).

Additionally, three participants experienced swelling; 2 in the MTA filling group and 1 in the GP and sealer group, which were pharmacologically managed. One case also showed discoloration in the MTA group.

## Discussion

Several factors, including the presence of preoperative pain and periapical lesions, a need for retreatment [26], irrigation solution, instrumentation techniques, and obturation methods and materials have been implicated in the level of postoperative endodontic pain [27]. The present trial demonstrated that the use of MTA followed by a coronal seal with GP as a root canal filling materials resulted in significantly less pain than the use of GP and sealer. Postoperative pain was evaluated for a short term since the pain rate and intensity have been shown to reach a maximum level a few days after the operation and lessen noticeably to a minimal level [28]. It has also been shown that premedication can decrease postoperative endodontic pain [29], so the participants who took medicine were not selected. Furthermore, a single-visit endodontic treatment was used to limit the effect of other variables, particularly the effect of intracanal medicaments and coronal leakage on postoperative pain [30].

The exact mechanism for lower pain in the MTA group is unknown; however, it may be attributed to the biocompatibility, sealing ability, hard tissue formation, and analgesic effect of the MTA. MTA is considered a non-mutagenic and non-neurotoxic compound without any side effects on microcirculation [14, 31]. Several investigations have shown that MTA induces anti-inflammatory effects, moderates the signaling molecules and cytokine output, and regulates cell attachment, proliferation, and differentiation, particularly in hard tissue-related cells [14, 32, 33].

MTA creates a bond with dentin via the induction of an interfacial layer, which enhances the push-out bond strength, bacterial-tight seal, and marginal adaptation and prevents fluid leakage [14, 31]. The release of calcium ions either induces hydroxyapatite or carbonates apatite over the entire surface and provides a biological seal. However, this is a long-term benefit of bioactivity [33]. It also has some antibacterial properties, particularly against facultative anaerobe bacteria, which is attributed to the alkalinizing activity and calcium hydroxide release [33, 34].

Bioceramic materials such as MTA also induce some analgesic effects and reduce nerve activity [4, 21]. An animal model showed that MTA injection did not excite the nociceptor action and had some analgesic effects similar to ketoprofen [4]. Electrophysiological evidence demonstrates the suppressive effects of MTA on the F1 neurons of suboesophageal ganglion by reducing the duration and intensity of the action potential and heightening the hyperpolarization amplitude [21]. This analgesic effect has been attributed to the outward flow of potassium ions in the cell membrane [21]. An in vitro study also revealed that the newly mixed and set form of some bioceramics did not activate the nociceptors and decreased the basal level of CGRP when exposed to the trigeminal sensory neurons directly [22]. Yet, there is no literature on humans to support a hypothesis that MTA offers analgesia, and future studies are required.

On the other hand, zinc washout, non-polymerized cytotoxic materials, and the substances released from the epoxy resin sealer might also cause pain [35]. A higher accumulation of inflammatory cells was shown to occur adjacent to AH Plus rather than MTA [36], and a fresh mix of AH Plus upregulated CGRP in trigeminal sensory neurons [37]. A clinical study revealed that analgesic intake was higher in the AH Plus group than in the iRoot SP sealer group [38]. A randomized clinical trial also concluded that a proper root canal instrumentation technique had a more critical effect on postoperative endodontic pain than the type of sealer. Notably, all evaluated sealers were calcium silicate-based cement [39].

The present trial revealed that the pain level after root canal treatment was significantly higher in females 6 and 12 h after the procedure. Some studies have shown that women are more prone to experience pain [39, 40], while other studies have not reported similar results [41]. However, the different eligibility criteria of the aforementioned studies and the different psychological and physiological responses to pain between genders should also be considered [40]. Differences in the reproductive organs, unstable hormonal levels, and higher psychosomatic diseases can lead to higher postoperative pain in females [42].

The pain level was similar for the maxillary and mandibular teeth in the present study. Some studies [43, 44] have reported no significant differences, while some others have reported a higher pain level in the mandibular teeth [27]. More important than tooth location is the type of tooth, as molar teeth are associated with a higher incidence of postoperative pain. It is believed that the higher complexity of root canal anatomy and more root canals may contribute to the higher incidence of pain in multi-rooted teeth [42]. Our study evaluated the level of pain in teeth with a single canal. Multiple canals are more prone to false-positive outcomes when sensibility tests are applied, due to more possibility of partial pulp necrosis, so the single-canal teeth were considered to eliminate the chance of misdiagnosis [45]. Future studies should examine the effect of other bio-ceramic as root canal filling materials on multi-rooted teeth.

A randomized controlled trial [15] has indicated that the success rate of MTA as a root canal filling material is comparable with that of GP. Several case reports describe the successful treatment of primary [46] and permanent teeth under different clinical situations particularly for challenging ones with using MTA as a root canal filling material [17, 18]. In addition, MTA as an endodontic sealer showed comparable outcomes to Tubliseal and AH Plus in a clinical study [19]. Hence, owing to comparable success rate, side effects (swelling), and lower postoperative pain, MTA can be a valuable option for clinicians.

Yet, drawbacks such as poor handling characteristics, difficult retrieval, discoloration, and low push-out and shear bond strength should be considered [20, 31]. Studies show that MTA has a higher tendency to discolor over time compared to other new bioceramics due to the presence of bismuth oxide and contamination with blood during the setting process [47–49]. It is recommended to avoid filling the coronal portion of the root canal with MTA and placing it in a dry root canal to prevent discoloration. Furthermore, the new bioceramics such as TotalFill and Biodentine exhibit higher push-out bond strength to dentin compared to MTA. This makes them valuable alternatives, particularly for use in the coronal region [50]. Finally, new bioceramics such as Biodentine, TotalFill, and PCM have been suggested as suitable alternatives, which allow the accomplishment of restorative procedures immediately after their placement [51], and their shear bond strength can be improved by using an additional hydrophobic bonding layer [52]. Accordingly, the potential of the new generation of bioceramics as a viable alternative should be evaluated.

To the best of our knowledge, the present investigation is the first clinical trial evaluating the pain caused by the MTA filling. However, it is important to interpret the results with caution as the study was conducted in an optimal clinical setting and individual cases may differ, particularly in terms of pulpal and periapical conditions. It is recommended that future clinical studies examine the use of a new generation of bioceramics as root canal filling materials on multi-rooted teeth in other clinical situations, such as vital pulp therapy. Additionally, further biological studies are suggested to better understand the effect of bioceramics on the mechanism of pain.

## Conclusions

Considering the lower postoperative pain of MTA as root canal filling materials it may be a valuable option for clinicians. However, the exact mechanism for lower pain levels remains unknown and further research are required.

### Electronic supplementary material

Below is the link to the electronic supplementary material.

Supplementary Material 1

Supplementary Material 2

## Data Availability

The data of this study are available from Azad University, but restrictions apply to the availability of these data due to ethical considerations. However, data are available from the corresponding authors upon reasonable request and with permission of The Azad University.

## Abbreviations

GP: Gutta-percha
MTA: Mineral trioxide aggregate
SD: Standard deviation
VSA: Visual Analog Scale
