# Supplementary material for: Postoperative pain of single-visit endodontic treatment with gutta-percha versus MTA filling: a randomized superiority trial
Source: BMC Oral Health. 2023 Dec 19;23:1026. doi: 10.1186/s12903-023-03372-6 (PMC10731764; doi:10.1186/s12903-023-03372-6)
Supplement: Supplementary file 1 — Supplementary Material 1 [file 12903_2023_3372_MOESM1_ESM.docx]

**Additional file 1**

**Table** S1. *Distribution of pain intensity (percent) based on time point*

|  | **Gutta-percha**  n=46 | | | | |  | **MTA**  n=48 | | | | |
| --- | --- | --- | --- | --- | --- | --- | --- | --- | --- | --- | --- |
|  | Time point | | | | |  | Time point | | | | |
| **Level of Pain (%)** | 6 h | 12 h | 24 h | 48 h | 72 h |  | 6 h | 12 h | 24 h | 48 h | 72 h |
| No | 36.0 | 42.0 | 56.0 | 64.0 | 70.0 |  | 68.0 | 84.0 | 90.0 | 90.0 | 92.0 |
| Mild | 32.0 | 30.0 | 22.0 | 14.0 | 12.0 |  | 22.0 | 6.0 | 4.0 | 6.0 | 4.0 |
| Moderate | 20.0 | 16.0 | 14.0 | 12.0 | 10.0 |  | 2.0 | 2.0 | 0.0 | 2.0 | 2.0 |
| Severe | 12.0 | 12.0 | 8.0 | 10.0 | 8.0 |  | 8.0 | 8.0 | 6.0 | 2.0 | 2.0 |
