# Supplementary material for: Postoperative pain of single-visit endodontic treatment with gutta-percha versus MTA filling: a randomized superiority trial
Source: BMC Oral Health. 2023 Dec 19;23:1026. doi: 10.1186/s12903-023-03372-6 (PMC10731764; doi:10.1186/s12903-023-03372-6)
Supplement: Supplementary file 2 — Supplementary Material 2 [file 12903_2023_3372_MOESM2_ESM.docx]

**Additional file 2**

**Figure S1**. The Flowchart of the experimental procedure
